# Social Media in der Anästhesiologie: ein systematischer Review zu Typisierung sowie Chancen und Risiken

**DOI:** 10.1007/s00101-026-01660-8

**Published:** 2026-03-06

**Authors:** Tobias Weigl, Mark Coburn

**Affiliations:** Klinik für Anästhesiologie und Operative Intensivmedizin, Universitätsklinikum Bonn, Venusberg-Campus 1, 53127 Bonn, Deutschland

**Keywords:** Medfluencer, Digitale Professionalität, Informationsqualität, Gesundheitskommunikation, Patientenaufklärung, Medical influencer, Digital professionalism, Information quality, Health communication, Patient education

## Abstract

**Hintergrund:**

Social Media hat sich global mit geschätzt zwischen 5,1 Mrd. und 5,5 Mrd. aktiven Nutzern verbreitet und findet auch in der Anästhesiologie eine zunehmende Bedeutung. Social Media ist mittlerweile fest etabliert als Wissens- und Suchmedium. „Medfluencer*Innen“ spielen in diesem Zusammenhang eine wachsende Rolle, indem sie gesundheitsbezogenes und fachdisziplinspezifisches Wissen verbreiten.

**Ziel:**

Ziel dieser Arbeit war es zum einen, eine Typisierung von „Medfluencer*Innen“ in der Anästhesiologie zu erlangen. Zum anderen sollten die Chancen und Risiken von Social Media für die Anästhesiologie herausgearbeitet werden.

**Methoden:**

Nach Formulierung der zwei zentralen Fragestellungen erfolgte eine systematische Literaturanalyse in der Datenbank PubMed. Relevante Inhalte der identifizierten Publikationen wurden in die Typisierung von „Medfluencer*Innen“ und/oder in Chancen und Risiken gebündelt. Die Literaturrecherche erfolgte systematisch und PRISMA-orientiert; die Evidenz wurde aufgrund der Heterogenität der Studiendesigns narrativ zusammengefasst.

**Ergebnisse:**

Insgesamt wurden 1727 mögliche Publikationen identifiziert und am Ende des Screeningprozesses 49 Artikel für diese Arbeit berücksichtigt (Originalarbeiten und Reviews). Es konnten 5 Typen von „anästhesiologischen Medfluencer*Innen“ mit unterschiedlichen Kompetenzen, Ressourcen und Zielen identifiziert werden. Es wurden jeweils 6 Chancen und Risiken von Social Media in der Anästhesiologie erkannt.

**Diskussion:**

Viralität ist kein Qualitätsmerkmal. Gleichzeitigt erzeugt Social Media Reichweite und damit einhergehend eine inhaltliche, ethische und rechtliche Verantwortung. Die Nutzung von Social Media in der Anästhesiologie bietet neuartige Möglichkeiten, verlangt aber eine kritische, evidenzbasierte und rechtskonforme Anwendung. Konkrete Lösungsansätze, um die Risiken zu minimieren, werden diskutiert und daraus Dos und Don’ts für „Medfluencer*Innen“ abgeleitet. Weitere Studien sind erforderlich, um den Einfluss auf konkrete anästhesiologische Maßnahmen sowie Auswirkungen für Patient*Innen ableiten zu können.

**Graphic abstract:**

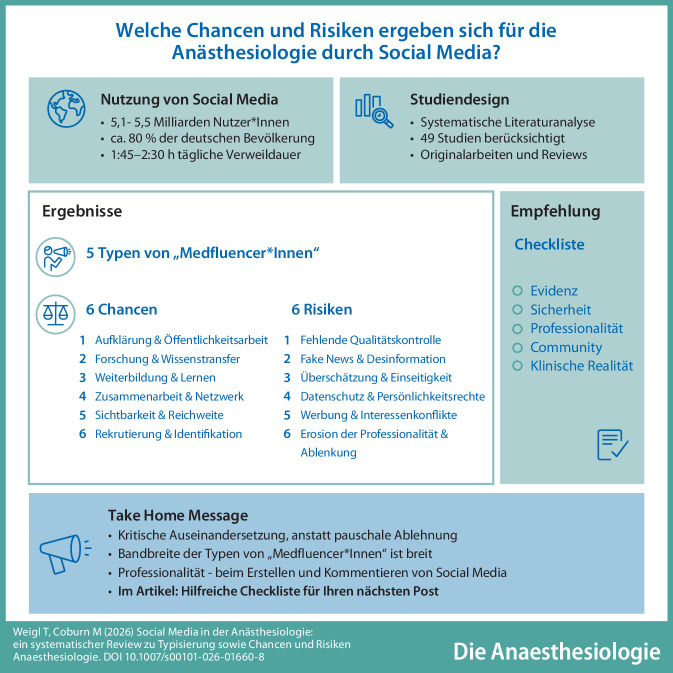

**Zusatzmaterial online:**

Die Online-Version dieses Artikels (10.1007/s00101-026-01660-8) enthält die Tabellen 6 und 7 als downloadbares PDF. Bitte scannen Sie den QR-Code.

## Abstract (en)

**Background:**

Social media have proliferated globally, with an estimated 5.1–5.5 billion active users and are gaining increasing relevance in anesthesiology. Social media are now firmly established as a medium for knowledge acquisition and information retrieval. In this context, “medfluencers” (individuals and institutions) play a growing role in disseminating health-related and discipline-specific information.

**Objective:**

The aim of this article is firstly, to develop a clearer understanding of “medfluencers” in anesthesiology and secondly, to synthesize the opportunities and risks of social media for anesthesiology through a structured literature analysis.

**Methods:**

Following specification of the two core research questions, a systematic literature search was conducted using predefined search strings in PubMed. Relevant contents from identified publications were synthesized into a typology of “medfluencers” and/or opportunities and risks of social media in anesthesiology. The literature search was performed in a systematic PRISMA-oriented manner and due to the heterogeneity of study designs and outcomes a narrative synthesis of the evidence was carried out.

**Results:**

A total of 1727 possible publications were identified and after the screening process 49 articles (original studies and reviews) were included. From the results five types of “medfluencers” in anesthesiology with distinct competencies, resources and aims were delineated. Social media activities in anesthesiology were synthesized into six opportunities and six risks for the respective “medfluencer” groups.

**Discussion:**

Virality is not a proxy for quality. Simultaneously, social media create a broad coverage and therefore entail content, ethical and legal responsibility. The use of social media offers novel possibilities for anesthesiology but requires a critical, evidence-based and legally compliant utilization. Concrete mitigation strategies to minimize risks are discussed, from which practical dos and don’ts for “medfluencers” are derived. Further studies are needed to be able to assess the effects on specific anesthesiological practices and the impacts on patient outcomes.

Social Media bietet in der Anästhesiologie neue Möglichkeiten für Wissensaustausch, Weiterbildung und Patientenaufklärung. Mittlerweile gibt es diverse Akteure mit unterschiedlichen Ressourcen und Zielen. Entscheidend für eine erfolgreiche Social-Media-Präsenz ist eine kritische und verantwortungsbewusste Nutzung zur Vermeidung von Fehlern und zur Reduktion immanenter Risiken. Es ist davon auszugehen, dass die Medizin und speziell auch die Anästhesiologie von Entwicklungen in diesem Feld beeinflusst werden und profitieren können.

## Hintergrund

Die Art und Weise, wie Menschen kommunizieren, Medien konsumieren und Informationen suchen, verändert sich. Weltweit und durch alle Altersklassen hinweg gehört Social Media für die Mehrheit der Menschen zum Alltag. Schätzungen zufolge gibt es ca. 5,1–5,5 Mrd. Nutzer*Innen [1, 2]. Im Jahr 2024 waren in Deutschland ca. 80 % der Gesamtbevölkerung aktive Social-Media-Anwender*Innen, davon ca. 84–92 % der unter 25-Jährigen (U 25) nahezu täglich [3, 4]. Die durchschnittliche Verweildauer beträgt zwischen 1:45 und 2:30 h pro Tag, wobei hier die Spanne von einigen Minuten bis über 8 h täglich reicht. Darüber hinaus bestehen altersbedingte, regionale und demografische Unterschiede [1, 4–6], s. Übersicht zu globalen und nationalen Social Media Kennzahlen in Tab. 1.

**Tab. 1:** Kennzahlen Social Media Nutzung 2024

Kennzahl	Wert	Definition	Monitoring-Berichte	Quelle
Globale Social-Media Nutzer	5,17 Mrd. (~64 % Weltbevölkerung)	„Aktive Nutzer*Innen“ = mind. einmal Monat aktiv	Statista 2025 [4]	https://de.statista.com/statistik/daten/studie/1479000/umfrage/nutzungsdauer-von-social-media-in-deutschland-nach-altersgruppen/
Deutschland, Nutzeranteil	80 % der Weltbevölkerung	„Aktive Nutzer*Innen“ = mind. einmal Monat aktiv	Statistisches Bundesamt 2024 [3]; DataReportal 2025 [2]	https://datareportal.com/reports/digital-2025-global-overview-report
Deutschland, U25, tägliche Nutzung	92 % der deutschen Bevölkerung	Tägliche aktive Nutzung	Statistisches Bundesamt 2024 [3]; Statista 2025 [4]	https://www.ard-zdf-medienstudie.de/
Ø Verweildauer, Social Media	105–150 min/Tag	Durchschnittliche tägliche aktive Nutzung Basierung auf Selbstauskunft, Erwachsene	ARD/ZDF Medienstudie 2025 [6]	https://www.ard-zdf-medienstudie.de/

Diente Social Media zu Beginn v. a. zur Unterhaltung und zum Austausch, so spielen Social-Media-Plattformen mittlerweile eine wichtige Rolle, um Antworten auf Fragen rund um die eigene Gesundheit sowie zu Ursachen, Anzeichen und Therapien von Krankheiten zu bekommen [1, 4, 7, 8]. Es ist davon auszugehen, dass unabhängig von Geschlecht, Alter und Bildung die Mehrheit der Patient*Innen schon einmal ihre Symptome sowie evtl. Fragen rund um die Anästhesiologie (z. B. Narkoseverfahren, Narkoserisiken) im Internet nachgeschaut hat und daraufhin auf Postings, Videos oder Blogbeiträge von „Medfluencer*Innen“ (engl. „medical influencer“) gestoßen ist.

Der Begriff „Medfluencer*In“ (Wortneuschöpfung aus *Medical* und *Influencer*) ist bislang nicht standardisiert bzw. definiert. Dieser Begriff wird jedoch zunehmend verwendet, um Personen oder Institutionen zu bezeichnen, die gesundheitsbezogene Inhalte über Social-Media-Plattformen öffentlichkeitswirksam verbreiten. In Deutschland gibt es nach aktuellen Schätzungen zwischen 800 und 1000 „Medfluencer*Innen“. Exakte Zahlen variieren je nach Definition [9, 10]. Zahlen zu „anästhesiologischen Medfluencer*Innen“ sind bisher nicht bekannt.

Schon früh wurde auf die Bedeutung von Social Media für die Anästhesiologie hingewiesen [11]. In den letzten Jahren, auch als Folge der COVID-19-Pandemie, ist das Interesse an den damit verbundenen Möglichkeiten gewachsen – sowohl national als auch international [12, 13]. Verschiedene Social-Media-Plattformen (z. B. YouTube, Instagram, X (ehemals Twitter) aber auch Podcast-Plattformen) haben sich zu einem Instrument für den Wissensaustausch, die berufliche Weiterbildung und die Vernetzung innerhalb der Anästhesiologie entwickelt [14, 15]. So sind beispielhaft die am häufigsten verwendeten anästhesiologischen Hashtags neben #anesthesia und #anaesthesia die Subthemen #intubation, #regionalanesthesia, #neuroanesthesia, und #cardiacanesthesia [16]. Die Deutsche Gesellschaft für Anästhesiologie und Intensivmedizin e. V. (DGAI), der Berufsverband Deutscher Anästhesisten (BDA) und auch Fachzeitschriften, wie z. B. *Die Anaesthesiologie*, nutzen Social Media als Kommunikationsmöglichkeit. Aus dieser rasanten Entwicklung der letzten Jahre sowie der Heterogenität der Literatur ergibt sich die Notwendigkeit eines systematischen Reviews. Ziel dieses systematischen Reviews ist es zum einen, eine Typisierung von „Medfluencer*Innen“ zu ermöglichen. Zum anderen soll auf Chancen und Risken durch aktive Social Media Postings hingewiesen werden.

## Methodik

Es wurde zunächst ein systematischer Review entsprechend den Kriterien der *Preferred reporting items for systematic reviews and meta-analyses* (PRISMA) [17] durchgeführt. Die Literaturrecherche erfolgte Mittels der PubMed-Datenbank für den Zeitraum 2020 bis Juni 2025. Es erfolgte eine Kombination von Titel/Abstract-Begriffen (TIAB) mit kontrollierten Schlagwörtern. Hierzu wurde ein Suchstring entwickelt, um die Trefferhäufigkeit mittels *MeSH („Medical Subject Headings“) terms* zu erhöhen. Die folgenden Suchbegriffe bzw. -kombinationen wurden verwendet: *„social media“ AND „anaesthesiology“*, *„social media“ AND „anaesthesia“,* *„anaesthesia“ AND „Twitter/Instagram/YouTube/TikTok“*, *„anaesthesiology“ AND „Twitter/Instagram/YouTube/TikTok“, „social media“ AND „health communication“*, *„medfluencer“ AND „health communication“*, *„public health communication“*, *„medical influencer“ *sowie „*medfluencer“*. Eingeschlossen wurden englisch- und deutschsprachige Originalarbeiten und Reviews. Siehe Infobox für die Suchstrategie in PubMed in der Literaturanalyse.

Nach Identifikation von relevanten Studien wurden zunächst PMID-basiert in einer My-NCBI-Collection die Duplikate entfernt. Anschließend wurden der Titel bezüglich der Ziele Typisierung von „Medfluencer*Innen“ sowie Chancen und Risiken von Social Media gescreent und im nächsten Schritt dann die Abstracts geprüft (TW). Im Anschluss wurden die ausgewählten Publikationen einem Volltextscreening zugeführt (TW). Ein Screening des Literaturverzeichnisses der eingeschlossenen Arbeiten wurde ebenfalls durchgeführt, und relevante Quellen wurden zusätzlich berücksichtigt. Aufgrund der heterogenen Studiendesigns und Outcomes wurde eine quantitative Synthese (Metaanalyse) nicht angestrebt; die Ergebnisse wurden im Sinne einer systematisch-narrativen Evidenzsynthese strukturiert zusammengefasst (Typisierung sowie Chancen und Risiken). Eine knappe Qualitätsevaluation der eingeschlossenen Studien wurde anhand von 6 Qualitätskriterien durchgeführt (Tab. 2).

**Tab. 2:** Qualitätsevaluationstabelle der inkludierten Studien

Qualitätskriterium	Erfüllungsgrad in den inkludierten Studien	Begründung
Publikationstyp und Peer-Review	Hoch	Es wurden ausschließlich Originalarbeiten und Reviews mit Peer-Review-Status eingeschlossen
Thematische Relevanz	Hoch	Studien ohne klaren Bezug zu Anästhesiologie oder medizinischen Social-Media-Kommunikation wurden konsequent ausgeschlossen
Sprachlicher Bias	Moderat	Die Beschränkung auf Deutsch und Englisch stellt eine Limitation (Sprachbias) dar
Aktualität der Daten	Hoch	Der Fokus liegt auf dem Zeitraum 2020 bis Juni 2025, was die aktuelle Dynamik des Feldes abbildet
Methodische Tiefe (Evidenzgrad)	Moderat bis niedrig	Die Literatur besteht fast ausschließlich aus Querschnittstudien; Langzeitdaten fehlen bislang
Wissenschaftliche Belegung	Heterogen	Die analysierten Social-Media-Inhalte belegen Aussagen nur selten durch Fachartikel oder Leitlinien

Ausgeschlossen wurden Publikationen, die keinen klaren Bezug zur Anästhesiologie oder keinen relevanten Bezug zu Social Media im Sinne medizinischer und gesundheitsbezogener Kommunikation haben. Ferner wurden Publikationstypen ohne Peer-Review oder ohne Primärevidenz ausgeschlossen, sowie wenn kein Volltext verfügbar und das Abstract nicht hinreichend zur Qualitätsbeurteilung war.

## Ergebnisse

**Abb. 1:**
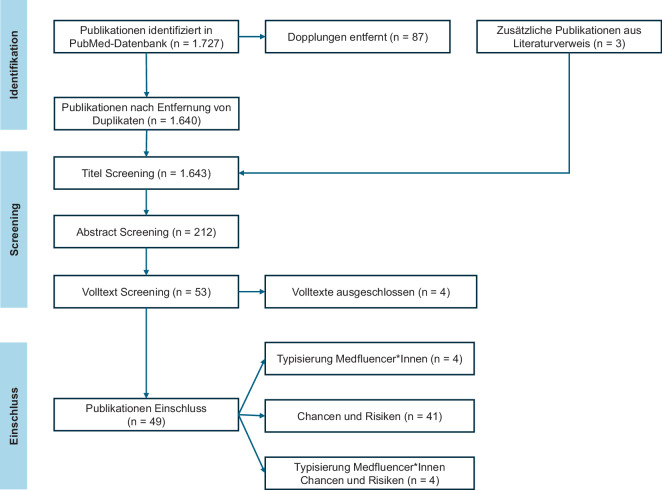
Einschlussdiagramm gemäß PRISMA

Insgesamt wurden 1727 mögliche Publikationen identifiziert und 87 Duplikate entfernt. Drei Publikationen wurden nach der Durchschau des Literaturverzeichnisses der eingeschlossenen Arbeiten zusätzlich berücksichtigt. Nach Screening von 1643 Titeln und der Prüfung von 212 Abstracts resultierten insgesamt 53 Publikationen, die einer Volltextanalyse zugeführt wurden. Von diesen wurden 4 Volltexte anschließend ausgeschlossen, da diese Texte keinen unmittelbaren Bezug zu den beiden Fragestellungen hatten. Es resultierten somit 49 inkludierte Publikationen, von denen 4 zur Typisierung von „Medfluencer*Innen“ (engl. *medical influencer*) und 41 Publikationen zur Bestimmung der Chancen und Risiken verwendet wurden. Vier Veröffentlichungen konnten für beide Fragestellungen verwendet werden. Abb. 1 zeigt das Einschlussdiagramm für diesen Review.

### Typisierung Medfluencer*Innen

Es lassen sich 5 Typen von „Medfluencer*Innen“ in der Anästhesiologie unterscheiden (Tab. 3), die sich nach Zielsetzung, Qualifikation und institutionellem Hintergrund unterscheiden. Die Spannbreite reicht von institutionellen professionell-medizinischen Kanälen von Fachgesellschaften oder Klinik-Accounts über approbierte Einzelpersonen bis hin zu Influencer*Innen ohne Ausbildung und/oder Berufserfahrung im Gesundheitsbereich bzw. speziell im Bereich der Anästhesiologie. Die fünfte Gruppe beinhaltet kommerziell-unternehmerisch Akteure, wie z. B. Medizintechnik-Hersteller. Dabei variieren sowohl die inhaltliche Tiefe als auch die Transparenz zu Qualifikationen und potenziellen Interessenkonflikten erheblich. Unabhängig vom Typ werden mehrheitlich von allen „Medfluencer*Innen“ die folgenden 5 inhaltlichen Schwerpunkte gesetzt:

1. Ursachen von Symptomen und/oder Erkrankungen,
2. typische Anzeichen und Symptome,
3. Tabuthemen und Mythen,
4. Therapieoptionen und -lösungen,
5. (Selbsthilfe‑)Tipps und praktische Hinweise.

**Tab. 3:** Typologie von Medfluencer*innen im Gesundheitswesen

Typ	Beispiele	Ziele (Beispiele)	Akteur*In	Qualifikation	Berücksichtigte Publikationen für den systematischen Review
Professionell-institutionell	Fachgesellschaft (z. B. DGAI), Klinik-Accounts	Aufklärung, Wissensvermittlung, Bekanntmachungen	Institution, Verband	Berufliche Erfahrung und Verantwortung im Gesundheitsbereich; meist im Team	Smailhodzic et al. 2021; Kirpekar et al. 2024 [32]; Marx et al. 2024 [23]; Ng et al. 2024; Van Ravenswaay et al. 2024 [67]
Professionell-individuell	Berufstätige Ärztinnen und Ärzte, berufstätige Pflegekraft	Edukation, Patientenbindung, (zusätzliche) Einnahmen	Einzelperson	Individuelle berufliche Erfahrung und Verantwortung	Guo et al. 2024; Kaňková et al. 2024; Kirpekar et al. 2024 [32]; Ng et al. 2024
Semiprofessionell-individuell	Medizinstudierende, Pflegekraft in Ausbildung	(Praktische) Einblicke, Wissensvermittlung, Peer-Kommunikation	Einzelperson	In Ausbildung, bisher kaum/keine Berufserfahrung	Smailhodzic et al. 2021; Ng et al. 2024; Van Ravenswaay et al. 2024 [67]
Persönlich-individuell	Gesundheits-Influencer*In, Patienten, Angehörige	Reichweite, Sichtbarkeit, Eigenwerbung	Einzelperson	Persönliche Erfahrungen und Meinung, ggf. (autodidaktisches) Selbststudium	Pourkarim et al. 2023 [9]; Marx et al. 2024 [23]
Kommerziell-unternehmerisch	Pharmaunternehmen, Medizintechnik Hersteller	Produktmarketing, Umsatzgenerierung, Branding, enge Konsumentenbindung	Unternehmen, Agentur	Markt- und Industrieexpertise	Pourkarim et al. 2023 [9]; Marx et al. 2024 [23]

**Abb. 2:**
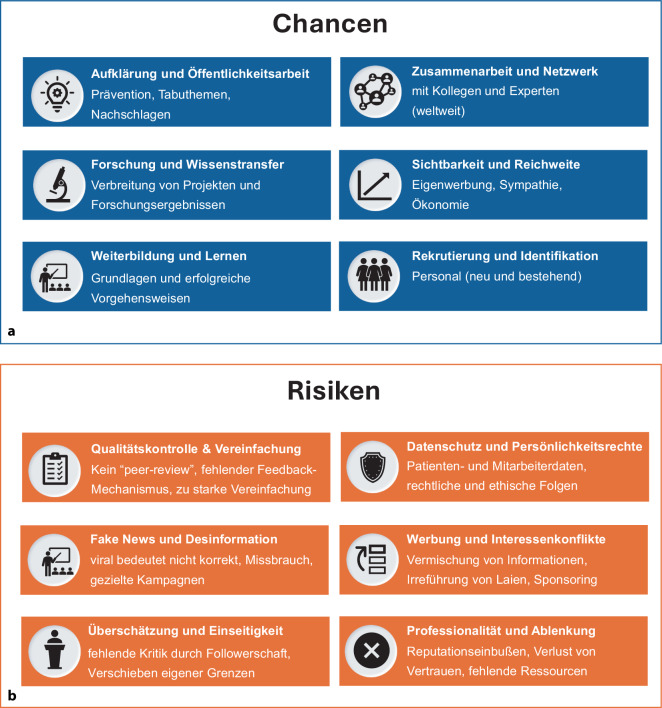
Chancen (**a**) und Risiken (**b**) für die Anästhesiologie durch Social Media

Chancen für die Anästhesiologie durch Social Media: Tab. 4 und Abb. 2a.

**Tab. 4:** Chancen und Risiken von Social Media in der Anästhesiologie

*Chancen*	*Beschreibung und Konsequenz*	*Berücksichtigte Publikationen für den systematischen Review*
Aufklärung und Öffentlichkeitsarbeit	Fördert Gesundheitskompetenz und Vertrauen der Patient*Innen	Harbell & Methangkool, 2021 [21]; Afful-Dadzie et al. 2023 [20]; Suarez-Lledo & Alvarez-Galvez 2021 [22]; Bamarni 2024 [8]; Marx et al. 2024 [23]; Nelms et al. 2024 [19]; Tan et al. 2024 [12]; Sablewski et al. 2025
	Resultat: Feedback-Mechanismus kann genutzt werden, um die Qualität der Patientenversorgung kontinuierlich zu verbessern und auf aktuelle Herausforderungen im medizinischen Alltag zu reagieren	
Forschung und Wissenstransfer	Hashtags, Peer-Gruppen und Plattformen ermöglichen internationalen Austausch und Forschungstransfer	Rong et al. 2020 [24]; Schwenk et al. 2020 [37]; Clavier et al. 2021; Erskine & Hendricks 2021 [27]; Jain et al. 2024 [15]; Nelms et al. 2024 [19]; Tan et al. 2024 [12]
	Resultat: sehr flexibel und global verfügbar, was zu einer breiteren Wissensverbreitung und schnelleren Wissensvermehrung führen kann	
Weiterbildung und Lernen	Flexible Weiterbildung (unabhängig von Ort und Zeit), individuelle Präferenzen, Lerngeschwindigkeit und Vorlieben	Nelsen et al. 2020 [30]; Tran et al. 2020 [33]; Cassai et al. 2021 [35]; Tulgar et al. 2023 [34]; Li et al. 2023 [29]; Ho et al. 2024 [28]; Jain et al. 2024 [15]; Rodrigues et al. 2024 [31]; Kirpekar et al. 2024 [32]; Marra et al. 2025 [36]
	Resultat: Sowohl für ärztliche Kolleg*Innen als auch (medizinisch-interessierte) Laien bieten Soziale Plattformen in Form von Videos, Bild- und Textbeiträge eine neue Form des Lernens und der Weiterbildung	
Zusammenarbeit und Netzwerk	Globale Vernetzung und Mentoring erleichtern Kooperationen, Lernen und Wissenstransfer, -neubildung	Schwenk et al. 2020 [37]; Clavier et al. 2021; Dunn et al. 2023 [38]; Ho et al. 2024 [28]; Jain et al. 2024 [15]; Kirpekar et al. 2024 [32]
	Resultat: neuartige grenzüberschreitende Zusammenarbeit innerhalb der eigenen Peer- und Mentoring-Möglichkeiten, die vor Ort möglicherweise nicht verfügbar sind	
Sichtbarkeit und Reichweite	Erhöhte Reichweite durch Konferenzen, Live-Tweets und digitale Sichtbarkeit	Schwenk et al. 2020 [37]; Dunn et al. 2023 [38]; Gomez et al. 2024 [39]; Mazzeffi et al. 2024; Tan et al. 2024 [12]
	Resultat: Bekanntheit, Teilnehmerzahlen und nationale sowie internationale Sichtbarkeit können um ein Vielfaches gesteigert werden	
Rekrutierung und Identifikation	Social Media kann die Arbeitgeberattraktivität und Teamkultur steigern	Feinstein et al. 2022 [40]; Dunn et al. 2023 [38]; Krisam & Altendorfer 2023 [42]; Plack et al. 2023 [41]
	Resultat: Aufbau eines relevanten und kostengünstigen Rekrutierungstools, dass einen Wettbewerbsvorteil gegenüber anderen Kliniken, Institutionen darstellen kann	
*Risiken*	*Beschreibung und Konsequenz*	*Berücksichtigte Publikationen für den systematischen Review*
Fehlende Qualitätskontrolle und Vereinfachung	Keine Peer-Review-Kontrolle, Verbreitung von Halbwissen möglich	Tran et al. 2020 [33]; Cassai et al. 2021 [35]; Suarez-Lledo & Alvarez-Galvez 2021 [22]; Borges do Nascimento et al. 2022 [43]; Tulgar et al. 2023 [34]; Gomez at al. 2024 [39]; Denniss & Lindberg 2025 [45]; Nickel et al. 2025 [44]
	Resultat: Nutzer*Innen werden (unabsichtlich) fehlinformiert und übernehmen solche Inhalte oft unkritisch, insbesondere wenn sie von vertrauenswürdigen Personen stammen	
Fake News und Desinformation	Untersuchungen zeigen gezielte Fehlinformationen auch im Bereich Anästhesie; v. a. während der COVID-19 Pandemie	Cassai et al. 2021 [35]; Gai et al. 2021 [46]; Tulgar et al. 2023 [34]; Kbaier et al. 2024; Rodrigues et al. 2024 [31]; Denniss & Lindberg 2025 [45]; Nickel et al. 2025 [44]; Sikosana et al. 2025 [47]; Stimpson et al. 2025 [48]
	Resultat: Unsicherheit und fehlendes Vertrauen der Patient*Innen in unsere medizinische Kompetenz und Arbeit	
Überschätzung und Einseitigkeit	Ärztliche Autorität führt zu Überschätzung und selektiver Wahrnehmung	Claessens et al. 2021; Tulgar et al. 2023 [34]; Indraccolo et al. 2025 [49]
	Resultat: Es besteht die Gefahr einer Überschätzung durch die medizinische Autorität und damit als Ergebnis eine verzerrte Wahrnehmung medizinischer Themen	
Datenschutz und Persönlichkeitsrechte	Unzureichende Anonymisierung kann Patientenrechte gefährden	Ahmed et al. 2020 [57]; Ghalavand et al. 2020 [54]; Pineau et al. 2023 [56]; Garmon et al. 2024 [53]; Van der Boon et al. 2024 [58]
	Resultat: Die Veröffentlichung sensibler Inhalte kann sowohl ethische Standards als auch rechtliche Vorgaben verletzen und (unerwartete) rechtliche Konsequenzen mit sich bringen	
Werbung und Interessenkonflikte	Unklare Kennzeichnung von Sponsorships; unzulässige Produktwerbung	Ghalavand et al. 2020 [54]; Cassai et al. 2021 [35]; Helou et al. 2023 [59]; Bamarni 2024 [8]; Gram et al. 2025
	Resultat: Verstöße können Rügen und Abmahnungen bis hin zu berufsrechtlichen Maßnahmen nach sich ziehen – sowohl für private als auch institutionelle „Medfluencer*Innen“	
Erosion der Professionalität und Ablenkung	Unbedachte Inhalte können das professionelle Ansehen schädigen	Ahmed et al. 2020 [57]; Helou et al. 2023 [59]; Tulgur et al. 2023; Bamarni 2024 [8]; Nelms et al. 2024 [19]; Tan et al. 2024 [12]; Garmon et al. 2024 [53]; Jain et al. 2024 [15]; Azer et al. 2025 [63]
	Resultat: Wenn diese Tätigkeiten nicht angemessen berücksichtigt und eingeplant werden, besteht Gefahr der Vernachlässigung der klinischen Kernaufgaben oder der Schaffung von zusätzlicher gesundheitlicher Risiken wie z. B. ein erhöhtes Burnout

Die identifizierte Literatur mit fünfundvierzig Publikationen wies eine Vielzahl an heterogenen Chancen und Risiken auf, die zu Schwerpunkten gebündelt und in 6 Chancen und 6 Risiken eingeteilt werden konnten.

1. **Aufklärung und Öffentlichkeitsarbeit**: Anästhesiolog*Innen können Social Media nutzen, um Patient*Innen über Anästhesieverfahren, Risiken und Vorteile zu informieren [12, 18, 19]. Dies trägt zur Erhöhung des allgemeinen Gesundheitsbewusstseins bei und kann das Vertrauen der Patient*Innen in die notwendigen anästhesiologischen Eingriffe stärken, häufige Patientenbedenken ansprechen und informierte (Patienten‑)Entscheidungen fördern. Ziel ist es, die Gesundheitskompetenz, die Selbstwirksamkeit und die Therapieadhärenz der Patienten*Innen zu erhöhen. Gleichzeitig kann Social Media den Anästhesiolog*Innen ein direktes Feedback von Kolleg*Innen und Patient*Innen ermöglichen [8, 20–23].
2. **Forschung und Wissenstransfer**: Social Media kann eine Plattform für den Austausch neuer Ideen und die Förderung von Forschungsprojekten bieten. Durch die Interaktion mit Kolleg*Innen und Forscher*Innen weltweit können neue Forschungsthemen identifiziert und interdisziplinäre Kooperationen initiiert werden. Fragen zu einem inhaltlichen Sachverhalt können niedrigschwellig einem internationalen Peer vorgestellt werden. Ferner ermöglicht Social Media eine schnelle und weitreichende Verbreitung von (anästhesiologischen) Forschungsergebnissen [12, 24, 25]. Durch Zugänge z. B. zu virtuellen Journal Clubs können aktuelle Forschungsergebnisse direkter, unkomplizierter und schneller ausgetauscht werden als in der Vergangenheit [15]. Weitere Untersuchungen konnten zeigen, dass wissenschaftliche Artikel, die über Social-Media-Plattformen verbreitet werden, um ein Vielfaches häufiger zitiert werden als solche, die keine Social-Media-Präsenz haben. Dies fördert nicht nur den Austausch von Wissen, sondern kann auch einen Einfluss auf die Verbreitung und Zitierung von Publikationen und damit auch auf den Impact-Faktor von Journals haben [24, 26, 27].
3. **Weiterbildung und Lernen**: Plattformen, wie YouTube und im gewissen Maße auch Instagram und TikTok, ermöglichen ein Nachschlagen, Informieren und (lebenslanges) Lernen. Praktische Videos zu Techniken und Interventionen am Patienten können jederzeit und überall abgerufen werden. Ein weiteres Medium zum Lernen und zur gezielten Weiterbildung können auch Podcasts sein, die auf Plattformen wie YouTube, Spotify oder anderen Podcast-Plattformen veröffentlicht werden. Durch Bildbeiträge, sog. Karusells, können auf Instagram, Facebook und weiteren sozialen Plattformen gezielt Erklärungen, Fakten, Hintergrundwissen u.v.m. zu anästhesiologischen Themen und Fragestellungen erstellt werden. Nutzer*Innen werden auf diese Weise auf anästhesiologische Themen aufmerksam, was über klassische Medien bisher vielleicht nicht (ausreichend) gelungen ist [15, 28–32]. Insbesondere in praktischen Anwendungsbereichen wie Intubation, Zugänge legen sowie Regionalanästhesie ermöglichen Social-Media-Beiträge, v. a. Videos, das Erlernen der richtigen Techniken und ein differenzierteres visuelles Verständnis [33–36]. Tab. 5 für 5 beispielhafte Podcast Accounts.
4. **Zusammenarbeit und Netzwerk**: Social Media ermöglicht es Anästhesiolog*Innen, sich weltweit mit Kolleg*Innen, Mentor*Innen und führenden Persönlichkeiten zu vernetzen. Solche (persönlichen) Netzwerke fördern den Austausch von Best Practices, Erfahrungen und Innovationen, die u. a. die Qualität der Patientenversorgung verbessern können. Ferner können spezielle Gruppen (offen oder geschlossen) gebildet werden, die explizit die Vernetzung und den Informationsaustausch innerhalb der eigenen Peers (Anästhesiolog*Innen) fördern [15, 25, 28, 32, 37, 38].
5. **Sichtbarkeit und Reichweite**: Krankenhäuser und speziell die anästhesiologischen Abteilungen/Kliniken können durch Social-Media-Aktivitäten die eigene Sichtbarkeit und Reputation ausweiten [12, 14, 38]. Social Media verbessert die Teilnahme und das Engagement bei medizinischen Konferenzen. Live-Tweets während Konferenzen ermöglichen es Anästhesiologen, wichtige Punkte aus Präsentationen und Workshops in Echtzeit zu teilen und so ein breiteres Publikum zu erreichen. Während virtueller Veranstaltungen halten Hashtags und Live-Diskussionen die Gemeinschaft verbunden und informiert. Dies erhöht die Reichweite solcher Veranstaltungen erheblich und ermöglicht es auch nichtanwesenden Fachleuten, von den Inhalten zu profitieren [37, 39].
6. **Rekrutierung und Identifikation**: Im Bereich der Medizin im Allgemeinen, und der Anästhesiologie im Speziellen, spielt Social-Media-Präsenz der eigenen Klinik und des eigenen Teams eine zunehmende Bedeutung zur Rekrutierung neuer (junger) Mitarbeiter*Innen. Einblicke in den Klinikalltag, die zu erwartenden Aufgaben und Informationen zu möglichen zukünftigen Arbeitskollegen spielen dabei eine Rolle [38, 40, 41]. Ein weiterer Aspekt ist die Förderung der Unternehmenskultur und -identifikation, z. B. durch Teilen gemeinsamer Erlebnisse [42].

**Tab. 5:** Deutschsprachige Anästhesiologie-Podcasts

Podcast (beispielhaft, alphabetisch sortiert)	Podcast (Institution, Person)	Erste Veröffentlichung	Anzahl, Folgen (zum 18.01.2026)
DGAI-Podcast	Deutsche Gesellschaft für Anästhesiologie und Intensivmedizin	01.08.2021	18
HAINS Talk	Klinik für Anästhesiologie, Universitätsklinikum Heidelberg	04.04.2022	52
Pin-up-docs – don’t panic (Hauptfolgen)	Thorben Doll, Johannes Pott (an niedersächsischen Schwerpunktversorgern)	15.01.2019	84
Team Timeout – Der Anästhesiepodcast	Klinik für Anästhesiologie und operative Intensivmedizin, Universitätsklinikum Bonn	06.09.2024	16
Young Urban Anesthesiologists (YUAN)	Klinik für Anästhesiologie, Universitätsmedizin Göttingen	03.04.2020	62

Risiken für die Anästhesiologie durch Social Media, Tab. 4 und Abb. 2b.

1. **Fehlende Qualitätskontrolle und Vereinfachung**: Im Gegensatz zu wissenschaftlichen Publikationen durch medizinische Fachzeitschriften gibt es auf Social Media keinen Review-Prozess. Dies kann dazu führen, dass Fehlinformationen ungefiltert weiterverbreitet werden. Zahlreiche Untersuchungen zeigen, dass ein signifikanter Anteil gesundheitsbezogener Posts unvollständig oder irreführend ist. Wissenschaftliche Studien und medizinische Innovationen sind oft komplex und nicht einfach in kurzen Social-Media-Beiträgen darstellbar. Die Verkürzung wissenschaftlicher Inhalte kann zu Missverständnissen führen oder Forschungsergebnisse aus dem Zusammenhang reißen [22, 39, 43–45]. Das Belegen von gesundheitsbezogenen Aussagen durch wissenschaftliche Fachartikel und/oder der Bezug zu Leitlinien o. Ä. findet nur selten statt [34, 35]. Social Media scheint die Verbreitung von „anekdotischer Evidenz“ zu fördern [33].
2. **Fake News und Desinformation**: Die Verbreitung von gezielten Falschinformationen ist eine wachsende Herausforderung auch im medizinischen Bereich – und auch in der Anästhesiologie. Während der COVID-19-Pandemie wurden gezielt Fehlinformationen über Anästhetika, Beatmungsverfahren und perioperative Risiken in sozialen Medien verbreitet [46]. Untersuchungen zeigen, dass gezielte Gesundheits-Missinformationen auf Plattformen weit verbreitet sind und in vielen Fällen durch die Sozialen Medien algorithmisch verstärkt werden [31, 34, 44, 45, 47]. Gezielte Desinformationskampagnen können das Vertrauen in das Gesundheitssystem und in die Medizin im Allgemeinen sowie auch in die Anästhesiologie untergraben bzw. erodieren [34, 35, 48].
3. **Überschätzung und Einseitigkeit**: Influencer*Innen, speziell auch „Medfluencer*Innen“, haben oft einen erheblichen Einfluss auf ihre Followerschaft, unabhängig von ihrer tatsächlichen fachlichen Expertise. Weil Ärzt*Innen in sozialen Medien häufig als Autoritäten wahrgenommen werden, kann ein sog. Halo-Effekt entstehen, bei dem ihre Aussagen auch in fachfremden Bereichen nicht hinterfragt werden. Der Halo-Effekt führt dazu, dass Expertise in einem Bereich (z. B. Anästhesiologie) automatisch auf andere Bereiche (z. B. Chirurgie, Virologie) übertragen wird – sowohl vom Publikum als auch von der Person selbst [49]. Ferner werden die Grenzen des eigenen Wissens immer mehr verschoben, und ein sog. Dunning-Kruger-Effekt [50] kann eintreten. Als Folge überschreiten „Medfluencer*Innen“ ihre eigenen (Wissens‑)Grenzen und äußern sich auch zu medizinischen Themen, die eigentlich nicht in deren Kompetenzbereich liegen – oftmals ohne es jedoch selbst zu erkennen [34, 51].
4. **Datenschutz und Persönlichkeitsrechte**: Datenschutz ist eine wesentliche Herausforderung, insbesondere, wenn Patientendaten oder klinische Fälle auf Social Media diskutiert werden. Auch auf Social Media muss die ärztliche Schweigepflicht (§ 203 Strafgesetzbuch) [52] berücksichtigt werden, und bereits wenige Kontextinformationen können Rückschlüsse auf Patient*Innen ermöglichen, was rechtliche und ethische Probleme nach sich ziehen kann [53, 54]. Daher sollten Einwilligungen ausdrücklich und dokumentiert vorliegen. Verstöße können straf- und berufsrechtlich relevant geahndet werden (dazu auch die europäische Datenschutz-Grundverordnung [55]). Untersuchungen von Social-Media-Beiträgen zeigen, dass ein nichtunerheblicher Anteil der Postings Verstöße gegen ärztliche Vertraulichkeit und in einigen Fällen sogar der Persönlichkeitsrechte der Patienten aufweisen [56, 57]. Darüber hinaus weisen aktuelle Studien darauf hin, dass ein Großteil der „Medfluencer*Innen“ Unsicherheiten im Umgang mit Datenschutzbestimmungen auf Social Media hat und diese Unsicherheit ein Risiko für ungewollte Verstöße darstellt [57, 58].
5. **Werbung und Interessenkonflikte**: Die Nutzung sozialer Medien durch „Medfluencer*Innen“ birgt ein hohes Risiko der Vermischung von Privat- und Berufsrolle, Marketing und Selbstdarstellung. Interessenkonflikte und Sponsorings werden auf Social-Media-Kanälen häufig unzureichend offengelegt. Dies unterminiert Transparenz, kann Laien in die Irre führen und widerspricht professionellen Standards (z. B., wenn Empfehlungs- oder Erfahrungsformate faktisch Testimonials darstellen) [8, 35, 54, 59, 60]. Im deutschen Kontext sind v. a. das Heilmittelwerbegesetz (HWG) sowie die berufsrechtlichen Regelungen der Bundesärztekammer maßgeblich [61, 62]. Konkret sind nach
  § 3 HWG irreführende Angaben (z. B. Übertreibungen, Heils‑/Erfolgsversprechen, Verharmlosung von Risiken) verboten,
  § 11 HWG Werbung mit Empfehlungen von im Gesundheitswesen Tätigen gegenüber Laien verboten. Vorsicht ist geboten bei „Testimonial-artigen“ Posts („Ich empfehle Präparat X“), Superlativen („die beste Narkosemethode“) sowie Vorher-Nachher-Bildern. Gleichzeitig fordern die Berufsordnungen eine sachliche, dem Gemeinwohl verpflichtete Information, die weder irreführt noch dem Ansehen des Berufsstandes schadet.
6. **Erosion der Professionalität und Ablenkung**: Für Social-Media-Beiträge von „Medfluencer*Innen“ sollten dieselben Standards der Professionalität wie im klinischen Alltag gelten. Verstöße gegen diese Grundsätze können nicht nur das Vertrauen in einzelne Ärzt*Innen, Pflegekräfte und Institutionen, sondern die gesamte Berufsgruppe beeinträchtigen [53, 57, 63]. Eine aktuelle Studie zeigt, dass bis zu 32 % der „Medfluencer*Innen“ sich nicht sicher sind, ob Inhalte auf ihren Social-Media-Konten als unprofessionell und inadäquat gelten würden [63]. Ein weiterer wichtiger Aspekt ist die professionelle Fokussierung auf die ursprünglichen Aktivitäten. Die ärztliche Tätigkeit von „Medfluencer*Innen“ darf nicht unter der zusätzlichen Tätigkeit auf den Sozialen Plattformen physisch und/oder psychisch leiden [12, 34]. Die aktive Nutzung von Social Media als „Medfluencer*In“ fordert eine regelmäßige Interaktion und Aktualisierung der Inhalte. Entsprechend investieren „Medfluencer*Innen“ oft erhebliche Zeit (und Geld) in die Planung, Erstellung und Moderation von Content-Beiträgen [8, 15, 59].

## Diskussion und Limitationen

In diesem systematischen Review konnten 5 Typen von „Medfluencer*Innen“ speziell im Bereich der Anästhesiologie (Privatpersonen als auch Institutionen) identifiziert werden. Zusätzlich konnten Chancen und Risiken, die sich durch die gezielte Erstellung von Social-Media-Beiträgen ergeben, in jeweils 6 Oberbegriffe gebündelt werden.

Eine einheitliche Definition von „Medfluencer*Innen“ gibt es nicht [9]. Basierend auf der Literaturauswahl haben wir für diesen systematischen Review „Medfluencer*Innen“ wie folgt definiert: Medfluencer*Innen sind im Gesundheitsbereich tätige Personen oder Organisationen, die über Social Media gesundheitsbezogene Inhalte publizieren und dabei durch Reichweite, Regelmäßigkeit und/oder fachliche Autorität Einfluss auf Gesundheitsverhalten oder Wissensstand von Laien und/oder Fachpublikum ausüben.

Ergänzend zu Qualifikation, Zielen und institutionellem Hintergrund stützen aktuelle Arbeiten die Typisierung entlang weiterer Achsen. So kann man zwischen mandatierten „Medfluencer*Innen“ (z. B. Fachgesellschaften, Klinik-PR, benannten Markenbotschafter*Innen) und selbstmandatierten „Medfluencer*Innen“ (individuelle Profile ohne offizielles Mandat) unterscheiden. Ein solches Mandat beeinflusst Erwartungshaltungen an Evidenztiefe, Verantwortlichkeit und Krisenkommunikation [14, 64]. Eine weitere Dimension der Typisierung könnte der Evidenzanker bzw. die Sicherstellung von Qualitätsmechanismen sein. Werden beispielhaft Leitlinien und Reviews zitiert, und gibt es z. B. fest etablierte Korrekturmechanismen (Erratum)? Diese Merkmale erlauben eine qualitätsbezogene Subtypisierung [44, 65]. Und schließlich ist auch eine Einteilung entsprechend der Monetarisierung möglich. Unterschieden werden nichtkommerzielle „Medfluencer*Innen“ (ohne Einnahmen), gemischt finanzierte (z. B. Werbeschaltungen, Spenden, Kursangebote) und kommerzielle Anbieter. Das Geschäftsmodell beeinflusst Themenwahl, Tonalität und potenzielle Bias-Risiken [10, 66].

Die Nutzung von Social Media in der Anästhesiologie bietet Chancen, verlangt aber gleichzeitig eine kritische, evidenzbasierte und rechtskonforme Anwendung. Nur, weil etwas viral geht, d. h. sehr oft aufgerufen und von Nutzer*Innen konsumiert wird, impliziert das nicht, dass dieser Beitrag auch inhaltlich korrekt sowie leitlinien- und rechtskonform ist. Viele „Medfluencer*Innen“ betreiben zunehmend nicht nur eine Social-Media-Plattform, sondern „recyceln“ den Content und veröffentlichen auf Instagram, YouTube, LinkedIn, TikTok und weiteren Plattformen. Diese Strategie der digitalen Omnipräsenz betreiben erfolgreiche Influencer*Innen bereits seit Jahren, und sie hat sich in den letzten Jahren zunehmend auch unter „Medfluencer*Innen“ etabliert. Entsprechend können Chancen und Risiken je nach Plattform variieren und die Komplexität bzw. Professionalität im Erstellen der Beiträge in Abhängigkeit der Plattform-Spezifika steigt [9, 32, 67]. Eine hohe Reichweite erzeugt Verantwortung, insbesondere vor dem Hintergrund, dass Ärztinnen und Ärzte einen Vertrauensvorschuss genießen und Aussagen häufig unkritisch von Nutzer*Innen bzw. Patient*Innen übernommen werden. Entsprechend sind reflektierte Nutzung, Transparenz und professionelle Standards zentral und sollten fest etabliert werden.

Die wachsende Rolle von „Medfluencer*Innen“ erfordert klare fachliche Zuständigkeiten und Kennzeichnung von Interessen. Ärztliche Inhalte unterliegen berufsethischen (z. B. Handreichung der Bundesärztekammer) und rechtlichen Rahmenbedingungen (z. B. HWG §§ 3, 11). Zusätzlich gilt auch in der digitalen Welt bzw. auf Social Media die ärztliche Schweigepflicht (§ 203 Strafgesetzbuch).

Im Folgenden finden Sie einen Vorschlag für Lösungsansätze, um insbesondere die aufgeführten 6 Risiken zu reduzieren.

1. **Fehlende Qualitätskontrolle und Vereinfachung: **Führen Sie einen internen 2‑Stufen-Review (Kolleg*Innencheck und Quellenprüfung) vor Veröffentlichung durch. Aussagen sollten immer mit Quellenangaben belegt werden, insbesondere (Patienten‑)Leitlinien sind zu empfehlen. Bei fachlichen Fehlern sollten die Inhalte ergänzt, korrigiert oder gelöscht werden. Etablieren Sie eine Korrekturstrategie mit raschem Erratum.
2. **Fake News und Desinformation: **Achten Sie nicht nur beim Erstellen, sondern auch beim Teilen von Content auf Korrektheit, d. h., orientieren Sie sich an Leitlinien und aktueller Studienlage. Argumentieren Sie mit Evidenz und nicht (ausschließlich) mit persönlicher Meinung. Darüber hinaus sollten Sie einen internen Umgang mit Kommentaren finden, z. B. Moderation und gezielte Beantwortung bzw. Richtigstellung von Falschaussagen.
3. **Überschätzung und Einseitigkeit:** Erstellen Sie eine klare Transparenz zu fachlicher Zuständigkeit und Kompetenzen. Markieren Sie Graubereiche auch als solche bzw. etablieren Sie sog. Co-Postings bzw. Co-Autorenschaft (z. B. Anästhesie und Infektiologie) bei inhaltlich herausfordernden Themen. Äußern Sie sich zu Themen, die zu ihrem Fach‑, Wissensbereich gehören und kennen Sie ihre Grenzen.
4. **Datenschutz und Persönlichkeitsrechte: **Achten Sie darauf, keine Patientendaten zu veröffentlichen. Fallvignetten sollten Sie nur bei informierter, dokumentierter Einwilligung und konsequenter Anonymisierung anwenden. Beachten Sie die rechtlichen Rahmenbedingungen gemäß HWG, § 203 StGB und sowie die Datenschutz-Grundverordnung der Europäischen Union (DSGVO).
5. **Werbung und Interessenkonflikte:** Benennen Sie Sponsoring und Kooperationen als solche klar. Generell sollte immer die Unabhängigkeit gewahrt bleiben, d. h., explizite Produktwerbungen sind zu unterlassen. Bei Kooperationen (auch bei sog. Disease-Awareness-Kampagnen) sollte explizit auf den Auftraggeber und das Sponsoring hingewiesen werden (z. B. durch Markierung mit *Sponsoring oder *Anzeige).
6. **Professionalität und Ablenkung:** Achten Sie auf eine klare Festlegung von Rollen und Zeiten. Ein Redaktions- bzw. Posting-Plan ist zu empfehlen, um Ressourcen und Verfügbarkeiten zu planen. Für Kolleg*Innen in Führungsposition könnte es sinnvoll sein, die eigenen Mitarbeiter*Innen bezüglich der Chancen und Risiken von Social Media zu schulen. Vielleicht sollte auch das Verhalten in sozialen Medien in professionellen Bewertungen bzw. Jahresgesprächen berücksichtigt werden.

Basierend auf den systematischen Review und den daraus vorgeschlagenen Lösungsansätzen fasst Tab. 6 die inhaltlichen, ethischen und rechtlichen Leitplanken als Dos und Don’ts bei der Erstellung von Social-Media-Beiträgen zusammen. Die Übersicht ersetzt keine Rechtsberatung, soll aber eine praktikable Orientierung bieten. Vor einem Social Media Posting sollte jede „Medfluencerin“, jeder „Medfluencer“ eine solche Übersicht/Checkliste durchgehen, um die Chancen für ein erfolgreiches Posting zu erhöhen und Risiken zu minimieren. Tab. 7 zeigt eine Pre-Post-Checkliste.

**Tab. 6:** Dos und Don’ts bei der Erstellung von Social Media Content

*Kategorie*	Dos (empfohlen)	Don’ts (vermeiden)
Transparenz und Qualifikation	Angabe von Titel, Institution und Fachgebiet	Vage oder irreführende Berufsbezeichnung
Quellenangabe und Evidenz	Zitieren aktueller Studien, Leitlinien, Reviews	Unbelegte Behauptungen ohne Referenzen; Meinung anstatt Evidenz
Claims und Versprechen	Hinweise auf Wahrscheinlichkeiten und Limitationen geben	Heilversprechen oder absolute Aussagen. Produkt-Versprechungen
Patientenbeispiele und Anonymisierung	Nur anonymisierte Fälle mit Einwilligung verwenden	Veröffentlichung identifizierbarer Daten oder Bilder ohne Zustimmung
Vertraulichkeit und Plattformwahl	Schweigepflicht beachten; Plattformen mit DSGVO-Konformität verwenden	Verwendung unsicherer Tools, Programme für vertrauliche Daten
Moderation und Kommentierung	Falschinformationen kommentieren oder berichtigen	Falschinformationen unkommentiert stehen lassen
Beratung vs. Information	Allgemeine Infos teilen, keine individuellen Empfehlungen; keine Produktwerbung	(Individuelle) Diagnosen oder Therapievorschläge ohne Untersuchung
Reflexion und Weiterbildung	Regelmäßige Anpassung an neue Standards und Richtlinien	Veraltete oder falsche Inhalte weiterverbreiten

**Tab. 7:** Die anästhesiologische Pre-Post-Checkliste

*1 Inhalts- & Qualitätscheck (Evidenz)*
– Evidenz statt Meinung: Basiert die Aussage auf aktuellen Leitlinien, Studien und Standards der Fachgesellschaft (z. B. DGAI) statt rein anekdotischer Evidenz?
– Quellenprüfung: Sind Quellen (z. B. PMID oder Links) für interessierte Leser*Innen angegeben?
– Vieraugenprinzip: Wurde der fachliche Content durch eine zweite qualifizierte Person (z. B. Oberarzt/Leitung) geprüft?
*2 Rechts- & Ethikcheck (Sicherheit)*
– Anonymisierung: Sind absolut keine Rückschlüsse auf Patient*Innen möglich (Ort, Zeit, seltene Fälle)?
– Einwilligung: Liegt bei Fallvignetten oder Fotos eine dokumentierte Einwilligung vor?
– Werberecht (HWG): Werden Heilsversprechen oder unzulässige Empfehlungen vermieden?
*3 Transparenz- & Rollencheck (Professionalität)*
– Interessenkonflikte: Sind Sponsoring oder Kooperationen klar markiert (z. B. #Anzeige)?
– Fachgrenzen: Bleibt der Inhalt des Beitrages innerhalb der fachlichen Kompetenz (Vermeidung fachfremder Aussagen)?
– Rollenklarheit: Ist klar ersichtlich, ob der Beitrag von einer Privatperson oder im Namen einer Institution erstellt wurde (Typisierung)?
*4 Interaktionscheck (Community)*
– Moderationsplan: Bestehen ausreichend Ressourcen, um auf fachliche Rückfragen zeitnah zu reagieren?
– Fehlermanagement: Existiert ein definierter Prozess für schnelle Korrekturen (Erratum) bei inhaltlichen Fehlern?
*5 Ressourcen- & Realitätscheck (Klinische Realität)*
– Zeitmanagement: Erfolgte die Content-Erstellung ohne Beeinträchtigung der klinischen Routineaufgaben oder der Patientensicherheit (keine Ablenkung im OP/auf der Intensivstation)?
– Institutionelle Compliance: Ist der Post mit den Social Media Guidelines bzw. Kommunikationsrichtlinien der Klinik oder des Arbeitgebers vereinbar?
– Transferprüfung: Wird klargestellt, dass gezeigte Techniken/Ideale unter den spezifischen Ressourcen der eigenen Person (Kompetenzen) oder Klinik (Personal, Ausstattung) abweichen können?

### Limitationen

Diese Übersichtsarbeit berichtet über den aktuellen Stand der Literatur zu Social Media in der Medizin, speziell in der Anästhesiologie. Dieses Feld ist sowohl in der Praxis als auch in der Forschung noch relativ neu und dynamisch. Dieser Umstand sowie die Vielfältigkeit dieses Gebiets (Kommunikation, Digitalisierung, Medizin) kann bedingen, dass nicht die gesamte relevante Literatur erfasst wurde. Neben einem Selektions‑/Publikationsbias ist auch ein sog. Sprachbias möglich, da ausschließlich englisch- und deutschsprachige Literatur verwendet wurde. Bei der Interpretation der Ergebnisse sind ebenfalls ein sog. Plattform‑/Algorithmus-Bias zu berücksichtigen. Social-Media-Plattformen bzw. deren Algorithmen priorisieren oft emotionalisierende und/oder vereinfachte Inhalte gegenüber komplexen Fachdiskussionen, was die Wahrnehmung der klinischen Realität verzerren kann. Zudem unterliegt die Literatur einem Survivorship-Bias: Gescheiterte oder durch Algorithmen marginalisierte Beiträge bleiben in der Auswertung und Literatur womöglich unterrepräsentiert, was die Generalisierbarkeit der Typisierung sowie der identifizierten Chancen und Risiken einschränken kann. Des Weiteren können aufgrund der Beschränkung auf den Suchzeitraum 2020 bis Juni 2025 relevante ältere Studien der systematischen Suche entgangen sein. Das Vorliegen von fast ausschließlich sog. Querschnittstudien führt zu einem Fehlen von Langzeitdaten.

## Schlussfolgerung

Sowohl in der Praxis als auch in der Wissenschaft sollte mehr Wissen über den gesundheitsförderlichen Einsatz von „Medfluencer*Innen“ gewonnen werden. Ferner sind weitere Aufklärung, Transparenz und zukünftige Regeln bedeutsam und ratsam. Mehr evidenzbasierte Forschung ist dafür notwendig, speziell auch bezüglich Verhalten der „Medfluencer*Innen“, Nutzen für Patient*Innen und Angehörige sowie konkreter (langfristiger) Implikationen und Auswirkungen auf unsere Fachdisziplin. Die Übertragbarkeit auf die klinische Realität stößt womöglich auf signifikante strukturelle Limitationen. Während die Literatur das Potenzial betont, scheitert die praktische Umsetzung in anästhesiologischen Kliniken oft an fehlenden Ressourcen (z. B. Zeit, Budget, Fachpersonal). Zudem erschweren (komplexe) Freigabeprozesse, mangelnde digitale Kompetenz der Mitarbeiter*Innen sowie mitunter strikte Compliance- und Datenschutzvorgaben eine agile Kommunikation.

### Infobox Suchstrategie in PubMed in der Literaturanalyse

( ( „Social Media“[Mesh] OR “social media”[tiab]) AND ( “Anesthesiology”[Mesh] OR “Anesthesia”[Mesh] OR anesthesiology[tiab] OR anaesthesiology[tiab] OR anesthesia[tiab] OR anaesthesia[tiab])) OR ( ( “Anesthesiology”[Mesh] OR “Anesthesia”[Mesh] OR anesthesiology[tiab] OR anaesthesiology[tiab] OR anesthesia[tiab] OR anaesthesia[tiab]) AND (Twitter[tiab] OR Instagram[tiab] OR YouTube[tiab] OR TikTok[tiab] OR “Internet”[Mesh] OR “Video Recording”[Mesh])) OR (( “Health Communication”[Mesh] OR “Communication”[Mesh] OR “health communication”[tiab]) AND ( “Social Media”[Mesh] OR “social media”[tiab])) OR ((medfluencer*[tiab] OR “medical influencer”[tiab] OR influencer*[tiab] OR “Opinion Leaders”[Mesh]) AND ( “Health Communication”[Mesh] OR “health communication”[tiab])) OR “public health communication”[tiab] OR ( “Public Health”[Mesh] AND “Communication”[Mesh])

AND ( „2020/01/01“[dp] : „2025/06/30“[dp])

AND (english[la] OR german[la])

AND (Review[pt] OR Systematic Review[pt] OR Meta-Analysis[pt] OR Journal Article[pt] OR Clinical Trial[pt] OR Observational Study[pt] OR Comparative Study[pt] OR Evaluation Study[pt] OR Multicenter Study[pt] OR Validation Study[pt] OR Qualitative Research[pt] OR Cohort Studies[Mesh] OR Case-Control Studies[Mesh] OR Cross-Sectional Studies[Mesh]) NOT (Editorial[pt] OR Letter[pt] OR Comment[pt] OR News[pt] OR Interview[pt] OR Guideline[pt] OR Practice Guideline[pt] OR Retracted Publication[pt] OR Retraction of Publication[pt])

## Fazit für die Praxis

- Die Bandbreite der Typen von „Medfluencer*Innen“ ist breit und eine Unterscheidung für die Nutzer*Innen, insbesondere für Patient*Innen schwierig. Eine plattformübergreifende Kennzeichnung und/oder Einordnung ist empfehlenswert.
- Ärztliche Professionalität gilt auch beim Erstellen und beim Kommentieren von Social-Media-Beiträgen. Dabei sind eine klare Benennung von Fachgrenzen, eine Kontextualisierung mit Studien/Leitlinien statt Argumentation mittels persönlicher Meinung sowie ein verantwortungsvoller Umgang mit sensiblen Patientendaten zu empfehlen.
- Eine ethisch reflektierte, kollegial abgestimmte Praxis verbindet digitale Sichtbarkeit mit Berufsethik, Patientensicherheit und Glaubwürdigkeit unserer Fachdisziplin.
- Wir empfehlen die Verwendung von konkreten Checklisten für die Erstellung von Social Media Beiträgen.

## Supplementary Information

ESM 1_Zusatzmaterial zum Beitrag

## Data Availability

Die während der aktuellen Studie generierten und analysierten Datensätze sind in diesem veröffentlichten Artikel enthalten oder auf begründete Anfrage beim korrespondierenden Autor erhältlich. The datasets generated and analysed during the current study are included in this published article or are available from the corresponding author on reasonable request.

## References

[1] wearesocial (2025) Digital 2025: Wie Deutschland Social Media nutzt – und was das für Brands bedeutet. https://wearesocial.com/de/blog/2025/02/digital-2025-wie-deutschland-social-media-nutzt-und-was-das-fuer-brands-bedeutet/. Accessed 11.10.2025

[2] DataReportal (2025) Digital 2025: Global Overview Report. https://datareportal.com/reports/digital-2025-global-overview-report. Accessed 11.10.2025

[3] Statistisches Bundesamt Erhebung über die private Nutzung von Informations- und Kommunikations- technologien. IKT 2024 (Mikrozensus-Unterstichprobe zur Internetnutzung). https://www.destatis.de/DE/Methoden/Qualitaet/Qualitaetsberichte/Einkommen-Konsum-Lebensbedingungen/ikt-private-haushalte-2024.html. Zugegriffen: 26. Okt. 2025

[4] Statista (2025) Nutzungsdauer von Social Media in Deutschland nach Altersgruppen im Jahr 2024. https://de.statista.com/statistik/daten/studie/1479000/umfrage/nutzungsdauer-von-social-media-in-deutschland-nach-altersgruppen/. Zugegriffen: 11. Okt. 2025

[5] Gaasly Social media trends in Germany 2024. https://www.gaasly.com/blog/social-media-trends-in-germany. Zugegriffen: 11. Oktober 2025

[6] ARD/ZDF ARD/ZDF-Medienstudie 2025. https://www.ard-zdf-medienstudie.de/. Zugegriffen: 11. Okt. 2025

[7] Rothfischer K (2021) Social Media - Key Opinion Leaders of the Future? J Eur CME 10:2014094 34912588 10.1080/21614083.2021.2014094 PMC8667906

[8] Bamarni A (2024) Ärzte auf Social Media – Einfluss auf die Arzt-Patienten-Kommunikation. Dermatologie (Heidelb) 10.1007/s00105-024-05391-y 38990339

[9] Pourkarim M, Nayebzadeh S, Alavian SM, Hataminasab SH (2023) Determination of Influencers’ Characteristics in the Health Sector. Hepat Mon In Press(In Press)

[10] Powell J, Pring T (2024) The impact of social media influencers on health outcomes: Systematic review. Soc Sci Med 340:116472 38070305 10.1016/j.socscimed.2023.116472

[11] George RB, Lozada MJ (2017) Anesthésiologistes, le temps est venu de mobiliser les réseaux sociaux! Can J Anaesth 64:1169–1175 28936589 10.1007/s12630-017-0976-z

[12] Tan JM, Simpao AF, Gálvez Delgado JA (2024) The Future of Social Media, Anesthesiology, and the Perioperative Physician. Anesth Analg 138:358–368 38215714 10.1213/ANE.0000000000006711

[13] Hong J, Siddique U, Echevarria G, Patel A, Lai YH, Pai BHP (2023) Cross-sectional study on utilisation of social media by regional anaesthesia and acute pain medicine fellowship programs in the United States. J Anaesthesiol Clin Pharmacol 39:571–576 38269162 10.4103/joacp.joacp_149_23 PMC10805201

[14] M M, L S, Z C, B M, E H, L K, R K, P M, N E (2024) Cross sectional study of Twitter (X) use among academic anesthesiology departments in the United States. PLoS One 19:e0298741 38330078 10.1371/journal.pone.0298741 PMC10852312

[15] Jain D, Doctor JR, Samantaray A, Ali Z (2024) Social media in anaesthesia education: Striking the right balance. Indian J Anaesth 68:317–319 38586270 10.4103/ija.ija_232_24 PMC10993949

[16] Gai N, Matava C (2019) Twitter Hashtags for Anesthesiologists: Building Global Communities. A A Pract 12:59–62 30102610 10.1213/XAA.0000000000000853

[17] Page MJ, McKenzie JE, Bossuyt PM, Boutron I, Hoffmann TC, Mulrow CD, Shamseer L, Tetzlaff JM, Akl EA, Brennan SE, Chou R, Glanville J, Grimshaw JM, Hróbjartsson A, Lalu MM, Li T, Loder EW, Mayo-Wilson E, McDonald S, McGuinness LA, Stewart LA, Thomas J, Tricco AC, Welch VA, Whiting P, Moher D (2021) The PRISMA 2020 statement: an updated guideline for reporting systematic reviews. BMJ 372:n71 33782057 10.1136/bmj.n71 PMC8005924

[18] A S, C E, M N, C M (2025) Preoperative Anxiety Management Practices in Pediatric Anesthesia: Comparative Analysis of an Online Survey Presented to Experts and Social Media Users. JMIR Pediatr Parent 8:e64561 39874201 10.2196/64561 PMC11790179

[19] Nelms MW, Javidan A, Chin KJ, Vignarajah M, Zhou F, Tian C, Lee Y, Kayssi A, Naji F, Singh M (2024) YouTube comme source d’éducation sur l’anesthésie périopératoire pour la patientèle et les stagiaires : une revue systématique. Can J Anaesth 71:1238–1250 38902576 10.1007/s12630-024-02791-5

[20] Afful-Dadzie E, Afful-Dadzie A, Egala SB (2023) Social media in health communication: A literature review of information quality. Health Inf Manag 52:3–17 33818176 10.1177/1833358321992683

[21] Harbell MW, Methangkool E (2021) Patient safety education in anesthesia: current state and future directions. Curr Opin Anaesthesiol 34:720–725 34817450 10.1097/ACO.0000000000001060

[22] Suarez-Lledo V, Alvarez-Galvez J (2021) Prevalence of Health Misinformation on Social Media: Systematic Review. J Med Internet Res 23:e17187 33470931 10.2196/17187 PMC7857950

[23] Marx J, Blanco B, Bollmann H (2024) Toward a Taxonomy of Social Media Influencers in Public Health Communication. In: Bui T (ed) Proceedings of the 57th Hawaii International Conference on System Sciences. Hawaii International Conference on System Sciences,

[24] Rong LQ, Lopes AJ, Hameed I, Gaudino M, Charlson ME (2020) Examining the correlation between Altmetric score and citation count in the anaesthesiology literature. Br J Anaesth 125:e223–e226 32571571 10.1016/j.bja.2020.04.086

[25] T C, E O, Z D, V C, B V, J S, E B (2021) The Association Between Professional Accounts on Social Networks Twitter and ResearchGate and the Number of Scientific Publications and Citations Among Anesthesia Researchers: Observational Study. J Med Internet Res 23:e29809 34652279 10.2196/29809 PMC8556638

[26] Thelwall M, Priem J, Eysenbach G (2011) Can Tweets Predict Citations? Metrics of Social Impact Based on Twitter and Correlation with Traditional Metrics of Scientific Impact. J Med Internet Res 13 10.2196/jmir.2012 PMC3278109 22173204

[27] Erskine N, Hendricks S (2021) The Use of Twitter by Medical Journals: Systematic Review of the Literature. J Med Internet Res 23:e26378 34319238 10.2196/26378 PMC8367184

[28] Ho V, Nguyen A, Kumar K (2024) Social media for learning: A qualitative exploration of the factors that impact clinical learners’ attitudes and intentions. The Clinical Teacher 21 10.1111/tct.13760 38494998

[29] Li C, Salman M, Esmail T, Matava C (2023) Use of Peer-Led Web-Based Platforms for Peer-Assisted Learning Among Canadian Anesthesia Residents and Fellows: Cross-Sectional Study. JMIR Form Res 7:e47977 37955954 10.2196/47977 PMC10682924

[30] Nelsen BR, Chen YYK, Lasic M, Bader AM, Arriaga AF (2020) Advances in anesthesia education: increasing access and collaboration in medical education, from E‑learning to telesimulation. Curr Opin Anaesthesiol 33:800–807 33060385 10.1097/ACO.0000000000000931

[31] Rodrigues F, Newell R, Rathnaiah Babu G RB, Chatterjee T, Sandhu NK, Gupta L (2024) The social media Infodemic of health-related misinformation and technical solutions. Health Policy and Technology 13:100846

[32] Kirpekar M, Kars MS, Mariano ER, Patel A (2024) The Professional Use of Social Media in Anesthesiology: Developing a Digital Presence Is as Easy as ABCDE. Anesth Analg 139:238–243 38367248 10.1213/ANE.0000000000006612

[33] Tran BW, Dhillon SK, Overholt AR, Huntoon M (2020) Social media for the regional anesthesiologist: can we use it in place of medical journals? Reg Anesth Pain Med 45:239–242 31719141 10.1136/rapm-2019-100835

[34] Tulgar S, Ahıskalıoğlu A, Thomas DT, de Cassai A, Gürkan Y (2023) Social Media Use Amongst Regional Anaesthesia and Pain Practitioners and Residents: Standardization and Ethical Considerations. Turk J Anaesthesiol Reanim 51:366–367 37587728 10.4274/TJAR.2023.231211 PMC10440485

[35] Cassai A, Geraldini F, Mariano ER, Kou A, Matava C (2021) Believe the hype? An evaluation of Twitter activity and publication trends related to the erector spinae plane block. J Clin Anesth 75:110499 34481365 10.1016/j.jclinane.2021.110499

[36] Marra A, de Siena AU, Iacovazzo C, Vargas M, Cesarano N, Collà Ruvolo C, Celentano G, Buonanno P (2025) Impact of YouTube® videos on knowledge on tracheal intubation for anesthesiologist trainees: a prospective observational study. J Anesth Analg Crit Care 5:12 40001208 10.1186/s44158-025-00232-3 PMC11854003

[37] Schwenk ES, Jaremko KM, Gupta RK, Elkassabany NM, Pawa A, Kou A, Mariano ER (2020) How Twitter conversations using hashtags #regionalanesthesia and #regionalanaesthesia have changed in the COVID-19 era. Reg Anesth Pain Med 45:765–766 32616566 10.1136/rapm-2020-101747 PMC7513259

[38] Dunn T, Patel S, Milam AJ, Brinkman J, Gorlin A, Harbell MW (2023) Influence of Social Media on Applicant Perceptions of Anesthesiology Residency Programs During the COVID-19 Pandemic: Quantitative Survey. JMIR Med Educ 9:e39831 37205642 10.2196/39831 PMC10337370

[39] Gomez K, Edwards HL, Kirby J (2024) Livestreaming clinical experience to remotely located learners: A critical narrative review. Med Educ 58:1032–1041 38606897 10.1111/medu.15392

[40] Feinstein MM, Schlosberg I, Da Shin W, Mercedes CR, Sison M, Katz D, Sherwin M (2022) Does residency program social media activity correlate with prospective applicant interest? J Clin Anesth 82:110959 36063741 10.1016/j.jclinane.2022.110959

[41] Plack DL, Abcejo AS, Kraus MB, Renew JR, Long TR, Sharpe EE (2023) Postgraduate-Year‑1 Residents’ Perceptions of Social Media and Virtual Applicant Recruitment: Cross-sectional Survey Study. Interact J Med Res 12:e42042 36943340 10.2196/42042 PMC10131859

[42] Krisam M, Altendorfer LM (2023) Influencer-Marketing im Gesundheitswesen: Eine Übersicht. Gesundheitswesen (Bundesverband der Ärzte des Öffentlichen Gesundheitsdienstes (Germany)) 85:100–102 33706391 10.1055/a-1377-6478

[43] Borges do Nascimento IJ, Pizarro AB, Almeida JM, Azzopardi-Muscat N, Gonçalves MA, Björklund M, Novillo-Ortiz D (2022) Infodemics and health misinformation: a systematic review of reviews. Bull World Health Organ 100:544–561 36062247 10.2471/BLT.21.287654 PMC9421549

[44] Nickel B, Moynihan R, Gram EG, Copp T, Taba M, Shih P, Heiss R, Gao M, Zadro JR (2025) Social Media Posts About Medical Tests With Potential for Overdiagnosis. JAMA Netw Open 8:e2461940 40009378 10.1001/jamanetworkopen.2024.61940 PMC11866028

[45] Denniss E, Lindberg R (2025) Social media and the spread of misinformation: infectious and a threat to public health. Health Promot Int 40 10.1093/heapro/daaf023 PMC11955583 40159949

[46] Gai N, So D, Siddiqui A, Steinberg BE (2021) Dissemination of Anesthesia Information During the Coronavirus Disease 2019 Pandemic Through Twitter: An Infodemiology Study. Anesth Analg 133:515–525 33886509 10.1213/ANE.0000000000005602

[47] Sikosana M, Maudsley-Barton S, Ajao O (2025) Analysing health misinformation with advanced centrality metrics in online social networks. PLOS Digit Health 4:e0000888 40522953 10.1371/journal.pdig.0000888 PMC12169528

[48] Stimpson JP, Park S, Adhikari EH, Nelson DB, Ortega AN (2025) Perceived Health Misinformation on Social Media and Public Trust in Health Care. Med Care 63:686–693 40793916 10.1097/MLR.0000000000002180 PMC12412888

[49] Indraccolo A, Brunetti R, Navarini C, Del Gatto C (2025) Moral virtues inferences: When limited information affects our attribution of virtues. Q J Exp Psychol (Hove) 78:2223–2234 39629667 10.1177/17470218241307652

[50] Kruger J, Dunning D (1999) Unskilled and unaware of it: how difficulties in recognizing one’s own incompetence lead to inflated self-assessments. J Pers Soc Psychol 77:1121–1134 10626367 10.1037//0022-3514.77.6.1121

[51] A C, O K‑P, F B, J R, G C, M F, G R, S C‑O, H R, V A, B M, A P, C P, Y‑E C (2021) Self-Illusion and Medical Expertise in the Era of COVID-19. Open Forum Infect Dis 8:ofab058 33880387 10.1093/ofid/ofab058 PMC8043259

[52] Bundesamt für Justiz Strafgesetzbuch § 203. https://www.gesetze-im-internet.de/stgb/__203.html. Zugegriffen: 11. Okt. 2025

[53] Garmon EH, Morris KC, McAllister RK (2024) Preserving medical professionalism in the age of social media. JCA Advances 1:100055

[54] Ghalavand H, Panahi S, Sedghi S (2020) Opportunities and challenges of social media for health knowledge management: A narrative review. J Educ Health Promot 9:144 32766329 10.4103/jehp.jehp_754_19 PMC7377150

[55] European Union Datenschutz Grundverordnung. https://eur-lex.europa.eu/legal-content/DE/TXT/?uri=CELEX%3A32016R0679. Zugegriffen: 11. Okt. 2025

[56] Pineau I, Pineau M, Selim J, Compère V, Besnier E, Zoé D, Popoff B, Clavier T (2023) Evaluation of Medical Confidentiality Breaches on Twitter Among Anesthesiology and Intensive Care Health Care Workers. Anesth Analg 137:418–425 37227950 10.1213/ANE.0000000000006540

[57] Ahmed W, Jagsi R, Gutheil TG, Katz MS (2020) Public Disclosure on Social Media of Identifiable Patient Information by Health Professionals: Content Analysis of Twitter Data. J Med Internet Res 22:e19746 32870160 10.2196/19746 PMC7492977

[58] van der Boon RMA, Camm AJ, Aguiar C, Biasin E, Breithardt G, Bueno H, Drossart I, Hoppe N, Kamenjasevic E, Ladeiras-Lopes R, McGreavy P, Lanzer P, Vidal-Perez R, Bruining N (2024) Risks and benefits of sharing patient information on social media: a digital dilemma. Eur Heart J Digit Health 5:199–207 38774369 10.1093/ehjdh/ztae009 PMC11104475

[59] Helou V, Mouzahem F, Makarem A, Noureldine HA, El-Khoury R, Al Oweini D, Halak R, Hneiny L, Khabsa J, Akl EA (2023) Conflict of interest and funding in health communication on social media: a systematic review. BMJ Open 13:e072258 37580091 10.1136/bmjopen-2023-072258 PMC10432670

[60] Gram EG, Moynihan R, Copp T, Shih P, Albarqouni L, Akl E, Smith C, Hardiman L, Nickel B (2025) Addressing misleading medical information on social media: a scoping review of current interventions. BMJ Evid Based Med 10.1136/bmjebm-2025-113704 PMC12703284 41047164

[61] Bundesamt für Justiz Heilmittelwerbegesetz. https://www.gesetze-im-internet.de/heilmwerbg. Zugegriffen: 11. Okt. 2025

[62] Bundesärztekammer (2023) Handreichung der Bundesärztekammer - Ärztinnen und Ärzte in sozialen Medien.

[63] Azer SA, AlShiha LZ, AlSkait GA, AlShayie RA, AlTamimi LA, Barasain RA, AlShaalan HS (2025) Use of social media and professional attitude and beliefs of medical students and interns: Should social media use be part of professional assessment? Medicine (Baltimore) 104:e43114 40660555 10.1097/MD.0000000000043114 PMC12262943

[64] Kington RS, Arnesen S, Chou W‑YS, Curry SJ, Lazer D, Villarruel AM (2021) Identifying Credible Sources of Health Information in Social Media: Principles and Attributes. NAM Perspectives 10.31478/202107a PMC8486420 34611600

[65] Bolderston A, Meeking K, Snaith B, Watson J, Westerink A, Woznitza N (2022) Five years of #MedRadJClub: An impact evaluation of an established twitter journal club. J of Medical Radiation Sci 69:165–173 10.1002/jmrs.569 PMC9163464 35143706

[66] Engel E, Gell S, Heiss R, Karsay K (2024) Social media influencers and adolescents’ health: A scoping review of the research field. Social Science Medicine 340:116387 38039770 10.1016/j.socscimed.2023.116387

[67] van Ravenswaay L, Parnes A, Nisly SA (2024) Clicks for credit: an analysis of healthcare professionals’ social media use and potential for continuing professional development activities. Med Educ Online 29:2316489 38359156 10.1080/10872981.2024.2316489 PMC10877644

