# Supplementary material for: Social media in anesthesiology: a systematic review on typology as well as opportunities and risks
Source: Anaesthesiologie. 2026 Mar 6;75(5):319–32. [Article in German] doi: 10.1007/s00101-026-01660-8 (PMC13128759; doi:10.1007/s00101-026-01660-8)
Supplement: Supplementary file 1 — ESM 1_Zusatzmaterial zum Beitrag [file 101_2026_1660_MOESM1_ESM.pdf]

Die anästhesiologische „Post-before-you-post"-Checkliste

**1. Inhalts- & Qualitätscheck (Evidenz)**
[ ] **Evidenz statt Meinung:** Basiert die Aussage auf aktuellen Leitlinien, Studien und Standards der Fachgesellschaft (z.B. DGAI) statt rein anekdotischer Evidenz?
[ ] **Quellenprüfung:** Sind Quellen (z. B. PMID oder Links) für interessierte Leser*innen angegeben?
[ ] **Vier-Augen-Prinzip:** Wurde der fachliche Content durch eine zweite qualifizierte Person (z. B. Oberarzt/Leitung) geprüft?
**2. Rechts- & Ethikcheck (Sicherheit)**
[ ] **Anonymisierung:** Sind absolut keine Rückschlüsse auf Patient*innen möglich (Ort, Zeit, seltene Fälle)?
[ ] **Einwilligung:** Liegt bei Fallvignetten oder Fotos eine dokumentierte Einwilligung vor?
[ ] **Werberecht (HWG):** Werden Heilsversprechen oder unzulässige Empfehlungen vermieden?
**3. Transparenz- & Rollencheck (Professionalität)**
[ ] **Interessenkonflikte:** Sind Sponsoring oder Kooperationen klar markiert (z. B. #Anzeige)?
[ ] **Fachgrenzen:** Bleibt der Inhalt des Beitrages innerhalb der fachlichen Kompetenz (Vermeidung fachfremder Aussagen)?
[ ] **Rollenklarheit:** Ist klar ersichtlich, ob der Beitrag von einer Privatperson oder im Namen einer Institution erstellt wurde (Typisierung)?
**4. Interaktionscheck (Community)**
[ ] **Moderationsplan:** Bestehen ausreichend Ressourcen, um auf fachliche Rückfragen zeitnah zu reagieren?
[ ] **Fehlermanagement:** Existiert ein definierter Prozess für schnelle Korrekturen (Erratum) bei inhaltlichen Fehlern?
**5. Ressourcen- & Realitätscheck (Klinische Realität)**
[ ] **Zeitmanagement:** Erfolgte die Content-Erstellung ohne Beeinträchtigung der klinischen Routineaufgaben oder der Patientensicherheit (keine Ablenkung im OP/ Intensivstation)?
[ ] **Institutionelle Compliance:** Ist der Post mit den Social-Media-Guidelines bzw. Kommunikationsrichtlinien der Klinik oder des Arbeitgebers vereinbar?
[ ] **Transferprüfung:** Wird klargestellt, dass gezeigte Techniken/ Ideale unter den spezifischen Ressourcen der eigenen Person (Kompetenzen) oder Klinik (Personal, Ausstattung) abweichen können?

Do’s und Don’t’s bei der Erstellung von Social Media Content

Kategorie	Do’s (empfohlen)	Don’ts (vermeiden)
**Transparenz und Qualifikation**	Angabe von Titel, Institution und Fachgebiet	Vage oder irreführende Berufsbezeichnung
**Quellenangabe und Evidenz**	Zitieren aktueller Studien, Leitlinien, Reviews	Unbelegte Behauptungen ohne Referenzen; Meinung anstatt Evidenz
**Claims und Versprechen**	Hinweise auf Wahrscheinlichkeiten und Limitationen geben	Heilversprechen oder absolute Aussagen. Produkt-Versprechungen
**Patientenbeispiele und Anonymisierung**	Nur anonymisierte Fälle mit Einwilligung verwenden	Veröffentlichung identifizierbarer Daten oder Bilder ohne Zustimmung
**Vertraulichkeit und Plattformwahl**	Schweigepflicht beachten; Plattformen mit DSGVO-Konformität verwenden	Verwendung unsicherer Tools, Programme für vertrauliche Daten
**Moderation und Kommentierung**	Falschinformationen kommentieren oder berichtigen	Falschinformationen unkommentiert stehen lassen
**Beratung vs. Information**	Allgemeine Infos teilen, keine individuellen Empfehlungen; keine Produktwerbung	(individuelle) Diagnosen oder Therapievorschläge ohne Untersuchung
**Reflexion und Weiterbildung**	Regelmäßige Anpassung an neue Standards und Richtlinien	Veraltete oder falsche Inhalte weiterverbreiten

Die Anaesthesiologie

Zusatzmaterial zum Beitrag **„Social Media in der Anästhesiologie: Ein systematischer Review zu Typisierung sowie Chancen und Risiken“** (2026) von Tobias Weigl und Mark Coburn in *Die Anaesthesiologie.*

Beitrag und Zusatzmaterial stehen Ihnen auf www.springermedizin.de zur Verfügung. Bitte geben Sie dort den Beitragstitel in die Suche ein.
